# Multiparametric MRI radiomics for the differentiation of brain glial cell hyperplasia from low-grade glioma

**DOI:** 10.1186/s12880-023-01086-3

**Published:** 2023-08-31

**Authors:** Siqian Gu, Jing Qian, Ling Yang, Zhilei Sun, Chunhong Hu, Ximing Wang, Su Hu, Yuyang Xie

**Affiliations:** 1 Department of Radiology, The First Affiliated Hosptial of Soochow University, 215006 Suzhou, China; 2 Soochow University, 215006 Suzhou, China

**Keywords:** Radiomics, Brain glial cell hyperplasia, Low-grade glioma, Multiparametric MRI images

## Abstract

**Background:**

Differentiating between low-grade glioma and brain glial cell hyperplasia is crucial for the customized clinical treatment of patients.

**Objective:**

Based on multiparametric MRI imaging and clinical risk factors, a radiomics-clinical model and nomogram were constructed for the distinction of brain glial cell hyperplasia from low-grade glioma.

**Methods:**

Patients with brain glial cell hyperplasia and low-grade glioma who underwent surgery at the First Affiliated Hospital of Soochow University from March 2016 to March 2022 were retrospectively included. In this study, A total of 41 patients of brain glial cell hyperplasia and 87 patients of low-grade glioma were divided into training group and validation group randomly at a ratio of 7:3. Radiomics features were extracted from T1-weighted imaging (T1WI), T2-weighted imaging (T2WI), diffusion-weighted imaging (DWI), contrast-enhanced T1-weighted imaging (T1-enhanced). Then, LASSO, SVM, and RF models were created in order to choose a model with a greater level of efficiency for calculating each patient’s Rad-score (radiomics score). The independent risk factors were identified via univariate and multivariate logistic regression analysis to filter the Rad-score and clinical risk variables in turn. A radiomics-clinical model was next built of which effectiveness was assessed.

**Results:**

Brain glial cell hyperplasia and low-grade gliomas from the 128 cases were randomly divided into 10 groups, of which 7 served as training group and 3 as validation group. The mass effect and Rad-score were two independent risk variables used in the construction of the radiomics-clinical model, and their respective AUCs for the training group and validation group were 0.847 and 0.858. The diagnostic accuracy, sensitivity, and specificity of the validation group were 0.821, 0.750, and 0.852 respectively.

**Conclusion:**

Combining with radiomics constructed by multiparametric MRI images and clinical features, the radiomics-clinical model and nomogram that were developed to distinguish between brain glial cell hyperplasia and low-grade glioma had a good performance.

## Introduction

Brain glial cell hyperplasia is a characteristic pathological process caused by the proliferation of glial cells under the stimulation of infection, poisoning, ischemia, hypoxia, trauma, ionizing radiation and other factors [1–3]. Despite being a repair reaction, excessive gliosis will impede neuronal structural repair and functional recovery [4–6], leading to a varaity of clinical symptoms.

Glioma is the most common malignancy of brain [7], which originates from normal glial cells [8]. Different grades and invasion features result in various treatment plans and prognoses. The primary issue in creating a treatment plan is preoperative grading, which also has an impact on the prognosis [9, 10]. Glial cell hyperplasia lacks distinctive clinical symptoms and imaging findings, and is frequently misdiagnosed as low-grade glioma, inflammation and other conditions [11]. Therefore, it is essential for clinical decision-making, therapy selection, and patient prognosis that brain glial cell hyperplasia and low-grade gliomas can be accurately distinguished before surgery.

MRI plays a key role in the identification of cerebral lesions given that it has the advantages of excellent soft tissue resolution, a clear anatomical backdrop, the absence of bone abnormalities, and three-dimensional imaging [12]. Radiomics extracts quantitative features contained in disease image data by mining data, and then detects disease image markers or predicts disease classification and grading. Radiomics’ advantages of being non-invasive, affordable, effective, and repeatable are beneficial for clinical decision-making [13–15].

This study aimed to further guide clinical decision-making through investigating the feasibility of multiparametric MRI radiomics in differentiating between low-grade gliomas and glial cell hyperplasia.

## Materials and methods

### Patients

**Fig. 1:**
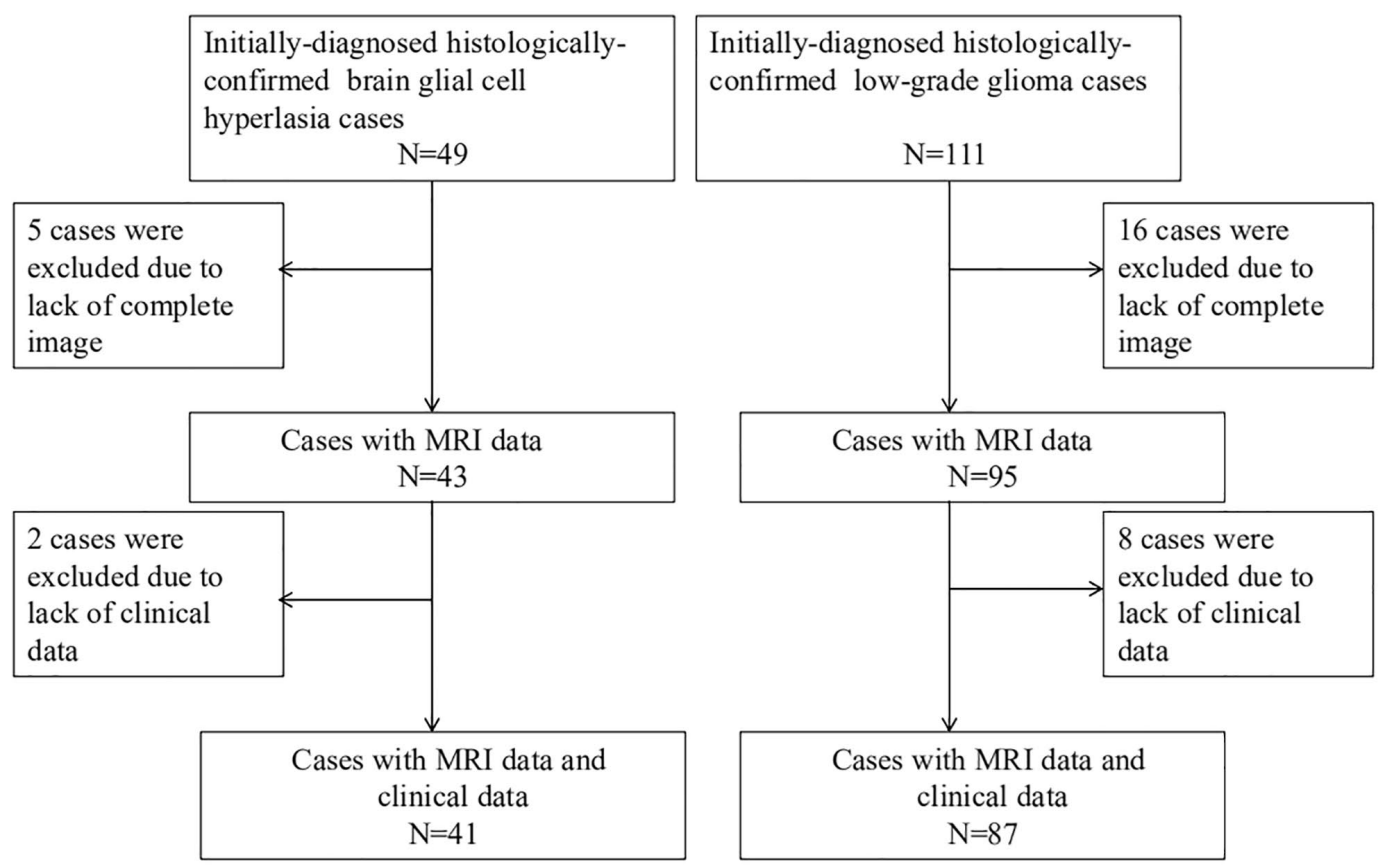
Shows the inclusion and exclusion criteria for 41 patients with glial cell hyperplasia and 87 patients with low-grade glioma

This retrospective study collected the clinical and imaging data of 41 patients with glial cell hyperplasia and 87 patients with low-grade glioma pathologically confirmed at the First Affiliated Hospital of Soochow University from March 2016 to March 2022. Both MRI plain and contrast-enhanced examination were performed on each patient. The inclusion criteria were: (1) Pathological data provided completely. (2) MRI plain and contrast-enhanced examination performed before surgical treatment. (3) No treatment given to interfere with tumor progression prior to examination. The exclusion criteria were: (1) Unqualified MRI scans. (2) Incomplete clinical data (Fig. 1). The age range for the glial cell hyperplasia group was 11 to 78 years (mean, 49.51 ± 17.55 years). Patients with pathologically confirmed low-grade glioma were randomly selected whose age ranged between 7 and 83 years, with a mean age of (53.74 ± 16.51) years.

### MR protocol

Images were obtained with 3.0T MRI system (Magnetom Skyra, Siemens Healthineers, Signa, GE Medical Systems). The patients should be instructed to maintain their heads motionless and, if required, to apply a gasket before the examination. The Siemens Skyra 3.0T MRI scanning parameters were as follows: T1WI: TE16ms, TR2170ms, thickness5mm, matrix256 × 256, T2WI: TE95ms, TR3400ms, thickness5mm, matrix384 × 384, DWI: TE87ms, TR4800ms, b = 1000s/mm2, thickness5mm, matrix168 × 180. The scanning settings after contrast injection were: TE2.26ms, TR2300ms, thickness1mm, matrix256 × 256. The GE 3.0T MRI scanning parameters were as follows: T1WI: TE28.25ms, TR:1962.20ms, thickness5mm, matrix512 × 512, T2WI: TE123.26ms, TR5100ms, thickness5mm, matrix512 × 512, DWI: TE75.10ms, TR5400.00ms, b = 1000s/mm2, thickness5mm, matrix256 × 256 mm. The scanning settings after contrast injection were: TE26.85ms, TR2693.36ms, thickness5ms, thickness512 × 512.

### Image analysis and region of interested segmentation

**Fig. 2:**
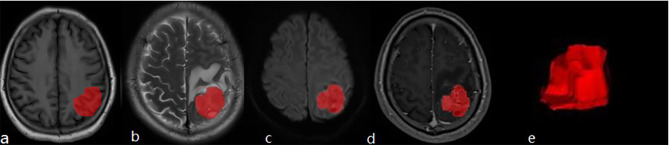
(**a**-**d**) The red areas showed the tumor level of T1WI, T2WI, DWI, T1-enhanced, (**e**) the generated tumor 3D-VOI

Two radiologists A and B (with experience of imaging diagnosis for 5 and 8 years, respectively) completed the image reading of T1WI, T2WI, DWI, T1-enhanced jointly without knowing the patients’ pathology results. Areas of tumor necrosis, cystic degeneration, and bleeding should be avoided when sketching. In case of disagreement, the two radiologists shall confer to reach a decision. The inter-class correlation coefficient (ICC) was then calculated with 30 lesions that were randomly chosen by the senior radiologist (Reader B). (1) ITK-SNAP (http://www.itksnap.org/pmwiki/pmwiki.php?n=Downloads.SNAP3) was used to manually segment the whole tumor lesion, and volume of interest (VOI) was defined along the tumor margin (Fig. 2). (2) Before feature extraction, the image was normalized and resampled to 3 mm×3 mm×3 mm voxel. Then FAE0.4.0 (https://github.com/salan668/FAE) was used to extract the ROI imaging features of tumor lesions. Based on the standardized images, 1781 radiomics features were extracted, including shape, first-order, gray-level cooccurrence matrix (GLCM), gray-level size zone matrix (GLSZM), gray-level dependence matrix (GLDM), and neighboring gray tone difference matrix (NGTDM). All extracted radiomics features are subjected to Mean Normanlization. The greatest linear correlation coefficient among all the characteristics in each T1WI, T2WI, DWI, and T1-enhanced model was screened out with the Person test for the subsequent analysis. (3) Texture feature selection: The included texture features were selected through the least absolute shrinkage and selection operator LASSO regression 10-fold cross validation method.

During sketching the tumor, maximum tumor diameter, tumor shape, mass effect, hemorrhage, cystic degeneration, necrosis, garland pattern enhancement, edema of the lesion were recorded at the same time.

### Construction of the radiomics prediction models

The LASSO, SVM and RF models were established following preprocessing of the extracted features based on the 10 times 7:3 random segmentation method. The AUC, accuracy, sensitivity, and specificity were applied to assess the performance. The precise procedure involved randomly splitting glial cell hyperplasia and gliomas into 10 portions, of which 7 were employed as the training group and 3 as the validation group. Each test can obtain the corresponding model prediction probability. The mean value of each prediction efficiency index was obtained as the final evaluation result of model efficiency after ten repetitions of 7:3 random split testing. Delong’s test was used to compare the AUC of the three models, then the radiomics model with highest efficiency was selected for further analysis.

### Radiomics-clinical model construction and performance evaluation

To obtain the regression coefficients, the optimal radiomics features were put into a logistic model. The Rad-score for each patient was subsequently calculated with a linear method based on the different weighting coefficient. Finally, the clinical features and Rad-score were filter out with logistics univariate analysis, the variables that showed statistically significant distinction were retained (P < 0.05), and logistics multivariate analysis was performed to determine the independent predictors (P < 0.05). The radiomics-clinical model for distinguishing glial cell hyperplasia from glioma was then constructed with these independent predictors. The performance could be evaluated through the calibration curve and AUC of training set and validation set. In order to further confirm the clinical value of the radiomics-clinical model, decision curve analysis was performed.

### Statistical analysis

The SPSS (25.0, IBM, Armonk, NY, USA) and R software (4.1.3:www.R-project.org) were applied for the statistical analysis. The Mann-Whitney U test and the Chi-square test were employed to determine whether there were statistically significant differences between in clinical features and imaging features.

## Results

### General clinical data

The study involved 128 patients, including 87 cases of low grade glioma and 41 cases of glial cell hyperplasia. Table 1 displays the patients’ clinical data and imaging findings.

**Table 1:** The clinical data and imaging findings of patients

Characteristic	glial cell hyperplasia	low grade glioma	χ^2^/z	p
n	41	87		
Gender, n (%)			0.040	0.841
Male	26 (63.4%)	52 (59.8%)		
Female	15 (36.6%)	35 (40.2%)		
Clinical symptoms, n (%)				1.000
Absent	3 (7.3%)	6 (6.9%)		
Present	38 (92.7%)	81 (93.1%)		
Tumor shape, n (%)			0.114	0.735
Irregular	19 (46.3%)	36 (41.4%)		
Regular	22 (53.7%)	51 (58.6%)		
Mass effect, n (%)			15.240	< 0.001
Absent	29 (70.7%)	28 (32.2%)		
Present	12 (29.3%)	59 (67.8%)		
Cystic degeneration, n (%)			4.473	0.034
Absent	33 (80.5%)	52 (59.8%)		
Present	8 (19.5%)	35 (40.2%)		
Hemorrhage, n (%)				0.241
Absent	34 (82.9%)	79 (90.8%)		
Present	7 (17.1%)	8 (9.2%)		
Necrosis, n (%)			2.526	0.112
Absent	36 (87.8%)	64 (73.6%)		
Present	5 (12.2%)	23 (26.4%)		
Garland pattern enhancement, n (%)			4.944	0.026
Absent	30 (73.2%)	44 (50.6%)		
Present	11 (26.8%)	43 (49.4%)		
Edema, n (%)			0.203	0.652
Absent	18 (43.9%)	33 (37.9%)		
Present	23 (56.1%)	54 (62.1%)		
Age, median (IQR)	51 (35, 63)	57 (42, 66)	-1.201	0.231
Maximum tumor diameter, median (IQR)	26 (18, 35)	34 (25.5, 45)	-3.191	0.001

### The establishment and evaluation of the imaging model

**Fig. 3:**
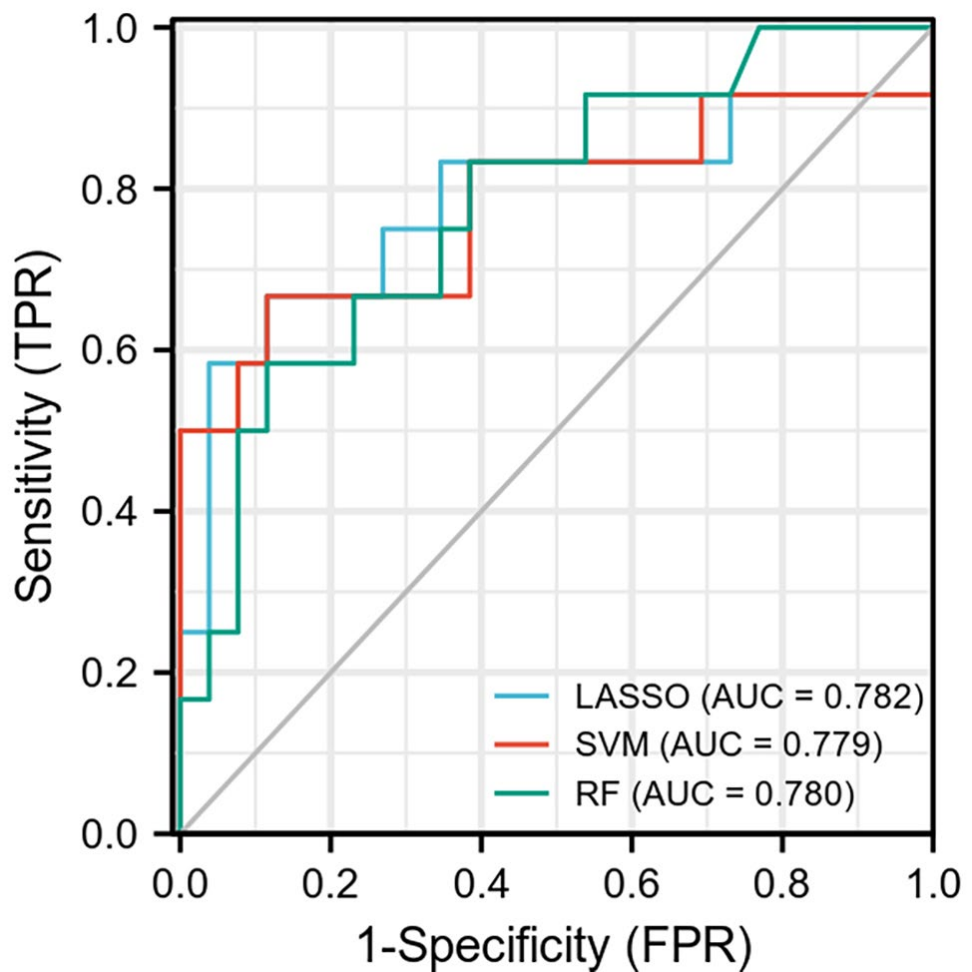
The ROC curve of the three radiomics model

**Fig. 4:**
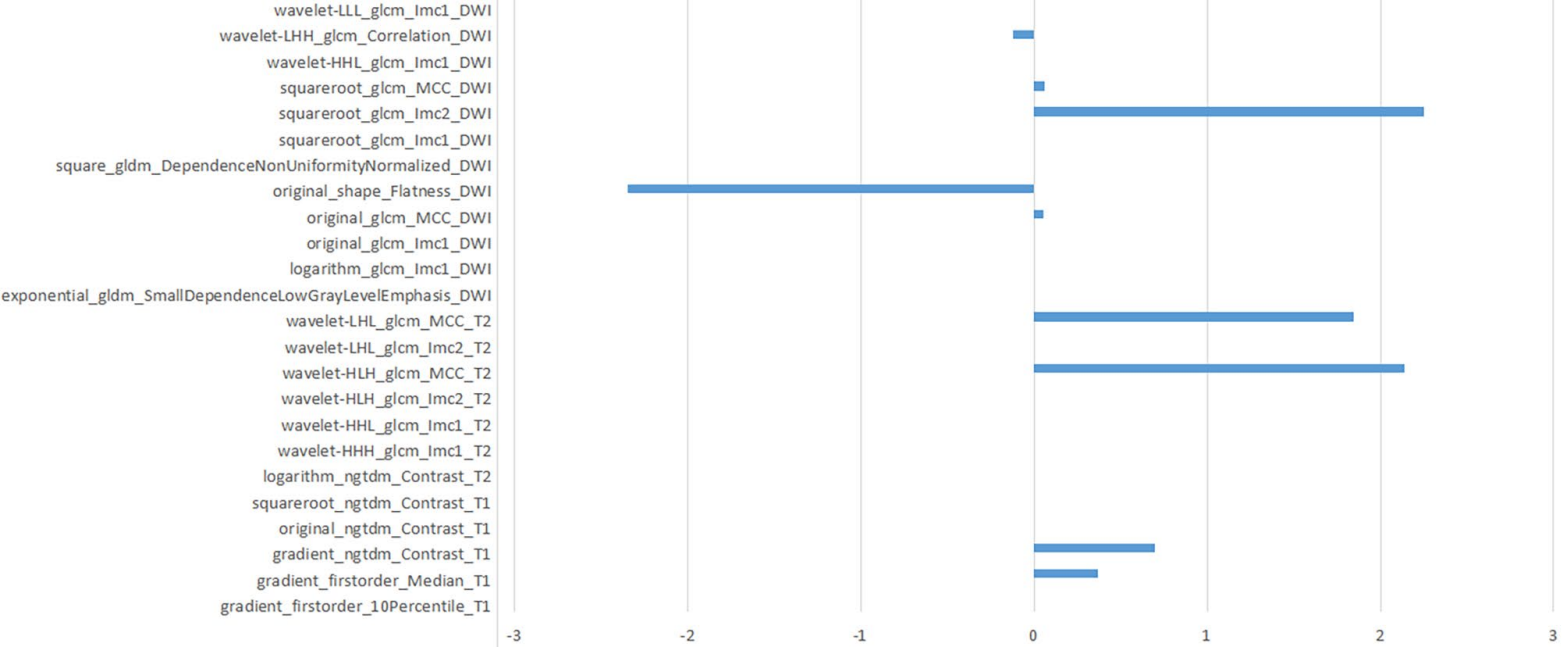
The 24 best feature information and the corresponding feature weight

The delineation results of both observers demonstrated good intergroup agreement, as determined by ICC analysis (ICC ≥ 0.85). Out of the 1781 characteristics in each model of T1WI, T2WI, DWI, and T1-enhanced, the Person-test maintained the 14 features with the highest linear correlation coefficient. Then the LASSO, SVM, and RF models were established, which AUCs for distinguishing between glial cell hyperplasia and low grade glioma were 0.782, 0.779, 0.780 (Table 2; Fig. 3). As is shown, the LASSO model, which was utilized to build the combined model, had the best predictive performance. Delong’s test indicated that there was no significant difference between the pairings of each model (P > 0.05) (Table 3). The LASSO model retained 24 optimal radiomics features, including two first-order, one shape, fifteen GLCM, two GLDM and four NGTDM (Fig. 4), which were linearly combined into one feature with the logistic model. The corresponding Rad-score is obtained with a Rad-score formula. Univariate and multivariate logistic regression analyses were used for calculating the Rad-score and clinical independent predictors, as depicted in Table 4. Mass effect (HR 3.674, 95% CI 1.011–13.359, P < 0.05) and Rad-score (HR 0.908, 95%CI 0.880 − 0.337, P < 0.05) were determined as two independent predictors.

**Table 2:** The comparison of the three model performance

Selection approach	Feature size	Cohort	AUC (95%CI)	Accuracy	Sensitivity	Specificity
LASSO24	24	Training	0.851(0.764–0.928)	0.778	0.759	0.787
		Validation	0.782(0.585–0.954)	0.816	0.667	0.885
SVM19	19	Training	0.845(0.748–0.934)	0.811	0.862	0.787
		Validation	0.779(0.586–0.955)	0.816	0.667	0.885
Random Forest20	20	Trianing	1.000(1.000–1.000)	1.000	1.000	1.000
		Validation	0.780(0.608–0.926)	0.790	0.583	0.885

**Table 3:** The results of Delong’s test

Variable 1	Variable 2	P
LASSO	SVM	0.933
LASSO	RF	0.981
SVM	RF	0.980

**Table 4:** Univariate and multivariate logistic regression analysis

Factors	Univariate		Multivariate	
	OR(95%CI)	P	OR(95%CI)	P
Gender	0.823(0.333–2.035)	0.673		
Age	0.994(0.968–1.020)	0.642		
Clinical symptoms	2.500(0.279–22.437)	0.413		
Maximum tumor diameter	**0.960(0.928–0.994)**	**0.020**	1.009(0.965–1.054) 0.698	
Tumor shape	0.693(0.285–1.684)	0.419		
Mass effect	**0.172(0.065–0.458)**	**< 0.001**	**0.243(0.068–0.865)**	**0.029**
Hemorrhage	3.717(0.959–14.406)	0.057		
Cystic degeneration	0.369(0.123–1.105)	0.075	0.917(0.276–3.044) 0.887	
Necrosis	0.325(0.086–1.220)	0.096		
Garland pattern enhancement	**0.369(0.142–0.960)**	**0.041**		
Edema	0.798(0.326–1.952)	0.622		
Rad-score	**1.121(1.069–1.176)**	**< 0.001**	**1.111(1.054–1.172)**	**< 0.001**

Bold suggests statistical significance at the level of P < 0.05. OR, odds ratio

### The establishment of nomogram and evalution

**Fig. 5:**
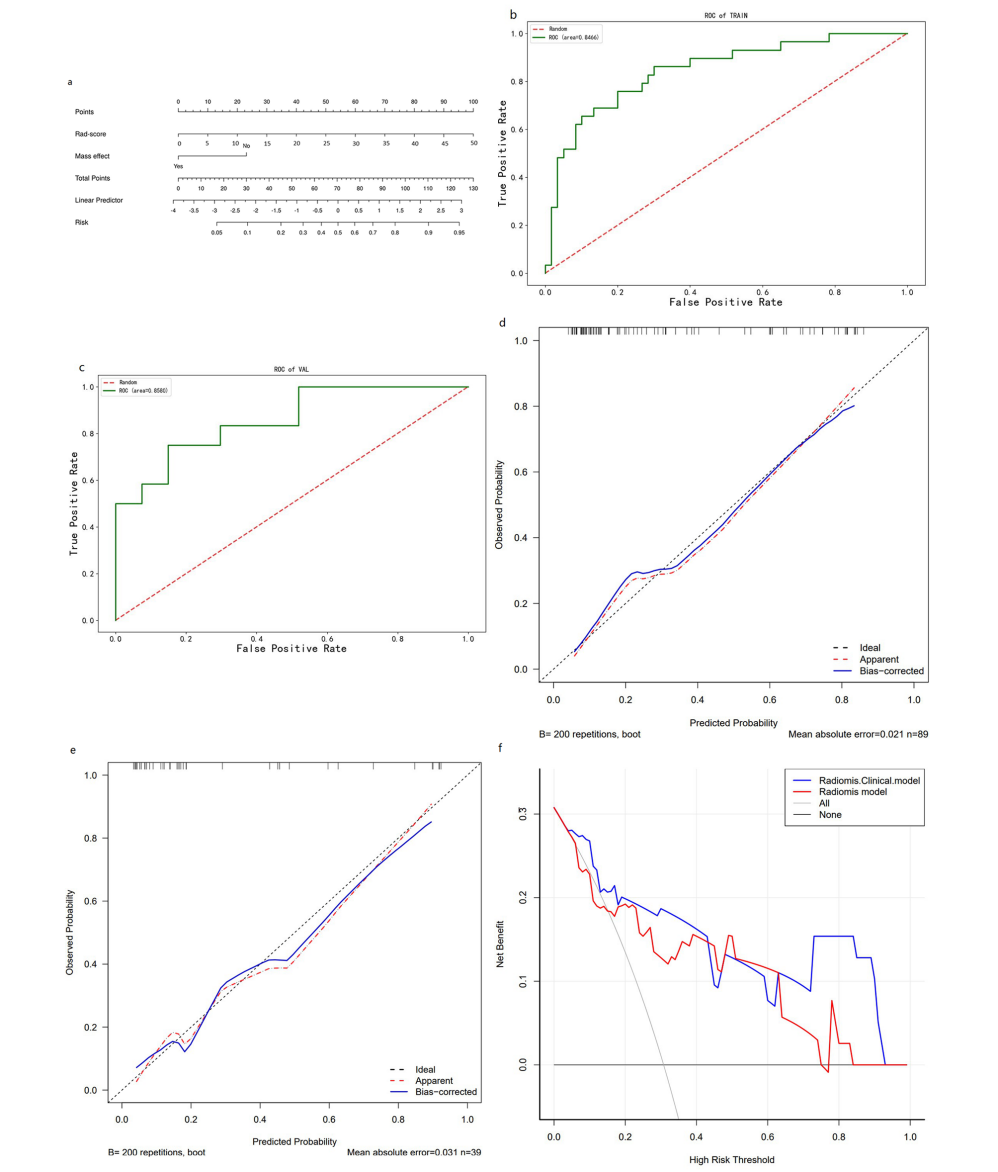
(**a**) is nomogram constructed based on combined model, (**b**) and (**c**) are the ROC curves of the training cohort and the validation cohort of the combined model, (**d**) and (**e**) are the calibration curves, (**f**) is the decision curve to differentiate glial cell hyperplasia from low-grade gliomas

Figure 5a shows the nomogram constructed with two independent predictors. The AUC, accuracy, sensitivity, and the specificity of the training group is 0.847, 0.764, 0.690, 0.800 respectively, compared to the validation group’s 0.858, 0.821, 0.750, and 0.852 (Fig. 5b, c). The H-L test results indicate that the radiomics-clinical model fits well (P = 0.987). In the training set and the validation, the calibration curve shows the good consistency of the prediction and the actual results (Fig. 5d, e). The decision curve (DCA) shows that the combined model has a higher overall net income and clinical decision effectiveness (Fig. 5f) .

## Discussion

Glial cell hyperplasia is a kind of intracranial benign lesion. Surgery is not necessary when the symptoms are mild, and follow-up can be possible [16]. Chemotherapy is typically unneeded in individuals who have apparent symptoms that require surgery, and their prognosis is better [17]. At the same time, some scholars believe that it is the early stage of the development of glioma and can evolve into glioma. Although the incidence is minimal, it has a bearing on how patients are treated and what their prognosis will be. Due to the potential for substantial neurological damage following blind surgery, preoperative illness detection is crucial. Glial cell hyperplasia and low-grade glioma are difficult to identify from one another due to the absence of distinctive clinical signs, which may result in unnecessary medical injury. The radiomics model may be effectively utilized to differentiate the tumor properties for patients who have lesions that are difficult to resect and are located in crucial functional regions. Therefore, it is of clinical significance to differentiate glial cell hyperplasia and glioma [18]. And radiomics can be used to distinguish the two. In this study, based on T1WI, T2WI, DWI, T1-enhanced image, the LASSO, SVM, and RF models were constructed by logistic regression algorithm, and the diagnostic efficacy was evaluated. Among the three models, the LASSO model shows the highest performance, with an AUC of 0.782, indicating that MRI images are feasible for the differentiation of glial cell hyperplasia and low-grade gliomas. The 24 optimal radiomics features were retained in the LASSO model, which are often difficult to be interpreted and analyzed visually but they can reflect the heterogeneity and complexity of the tumor microenvironment [19], Shape_Flatness represents the relationship between the largest and smallest principal components in the ROI shape, which shows difference because gliomas are malignant tumors with considerable heterogeneity. Dependence non uniformity normalized refers to the similarity of adjacent voxels in the entire image, which the lower the value, the higher the homogeneity between adjacent voxels. The joint distribution of two pixels with a certain spatial position relationship is described by GLCM and may be linked to variations in cell origin [20]. Firstorder reflects voxel intensity distribution in the image region, NGTDM quantifies the sum of differences between the gray level of a pixel or voxel and the average gray level of its neighboring pixels or voxels within a predefined distance. Even though these features are difficult to be identified by the naked eye, radiomics can fully exploit these traits, which can provide significant information for the diagnosis and prediction of diseases [21].

In this study, clinical features were combined to establiesh a radiomics-clinical model to explore whether the combination of clinicopathological risk factors may improve the prediction accuracy of glial cell hyperplasia. Recent studies have revealed that diagnosis of glioma is highly related to the presence or absence of clinical symptoms, regular shape, mass effect [22], cystic degeneration, hemorrhage, necrosis, garland pattern enhancement [23], edema, and maximum tumor diameter [24]. The differentiation of glial cell hyperplasia and low-grade glioma was shown to be strongly correlated with mass effect and Rad-score by the findings of univariate and multivariate logistic regression analysis. Mass effect and Rad-score are independent predictors for glioma. Gliomas develop rapidly and the mass effect is more obvious. Glial cell hyperplasia is a benign intracranial lesion and develops slowly. Since gliomas are extremely cancerous, they typically require postoperative radiotherapy and chemotherapy [25]. The prognosis of glial cell hyperplasia is better. In our study, only 5 cases of glial cell hyperplasia were treated with radiotherapy and chemotherapy, while 42 cases of glioma were treated with radiotherapy and chemotherapy, demonstrating the clinical importance of being able to differentiate between the two. The radiomics-clinical model, which was constructed via mass effect and Rad-score, was superior to the single radiomics model in the validation group, and the AUC of the validation set was 0.858. Finally, the presentation of the nomogram showed improved prediction accuracy. The calibration curve further proved the high consistency between the predicted probabilities and pathological result. The decision curve indicated that the combined model could achieve higher clinical decision effectiveness than the radiomics model, which could support clinical precision care and follow-up of patients with glial cell hyperplasia.

However, there are several limitations to our study. First, considering that the study was retrospective, a selective bias in a small group of patients from a single institution might have existed. Second, the sample size is relatively small and thus data from multiple centers are required to detect the overall performance of the model from external validation. In addition, the tumor ROI was drawn manually, which may lead to some errors. In the future, the lesions will be automatically segmented with the deep learning technique.

In conclusion, our results have been demonstrated that the radiomics features extracted from MRI, combining important clinicopathological risk factors, are of great significance in differentiating glial cell hyperplasia from low-grade glioma at an early stage, and can assist in the formulation of diagnosis and treatment individually.

## Data Availability

All data generated or analysed during this study are included in this published article.
